# Supplementary material for: Nutraceuticals Synergistically Promote Osteogenesis in Cultured 7F2 Osteoblasts and Mitigate Inhibition of Differentiation and Maturation in Simulated Microgravity
Source: Int J Mol Sci. 2021 Dec 23;23(1):136. doi: 10.3390/ijms23010136 (PMC8745420; doi:10.3390/ijms23010136)
Supplement: Supplementary file 1 [file ijms-23-00136-s001.zip › ijms-1515595-supplementary.pdf]

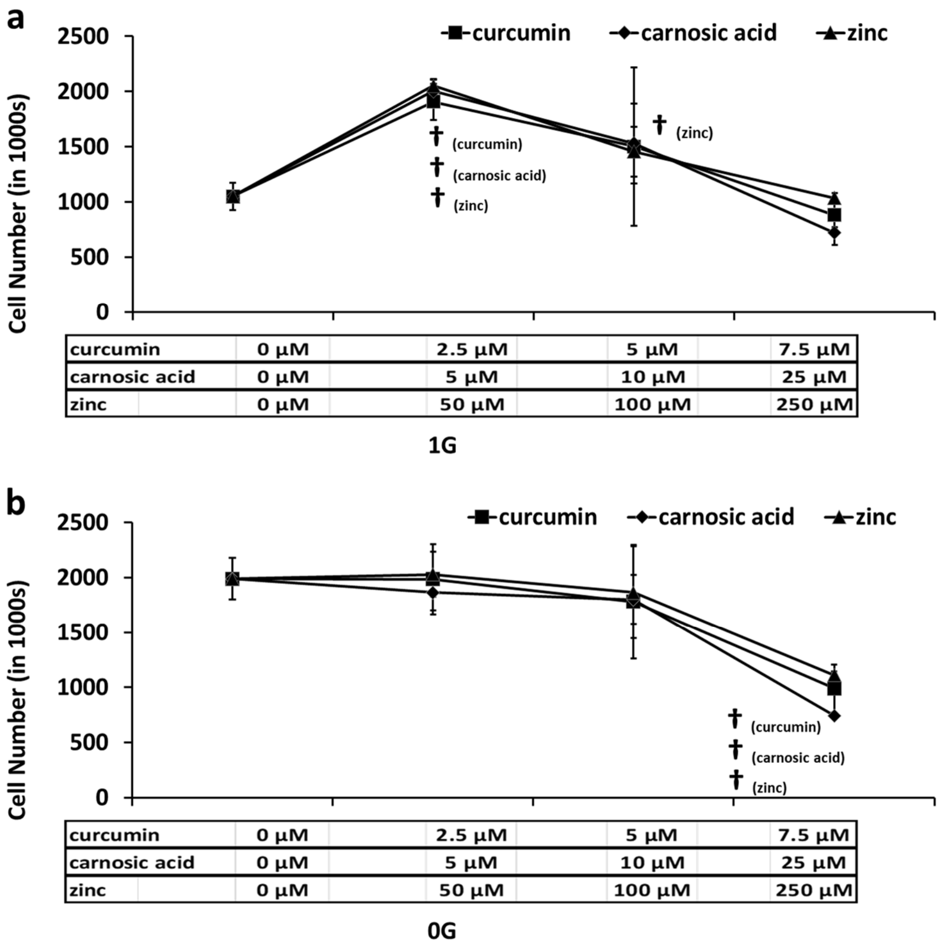

Supplemental Figure S1. Effects of nutraceuticals on total cell numbers, as quantified by PICO green after 6 days (n = 3) in a T-12 flask with an assayed surface area of 6.25 cm^2 (half of the flask). (**a**) 1G (Earth) profiles; (**b**) SMG (simulated microgravity) profiles. **Cross** (†) indicates a significant difference $p < 0.05$ versus 0 μM control. The results reflect means ±S.D from three independent experiments (n = 3 biological replicates). Statistical analysis was conducted using ANOVA followed by Tukey's post-hoc test.

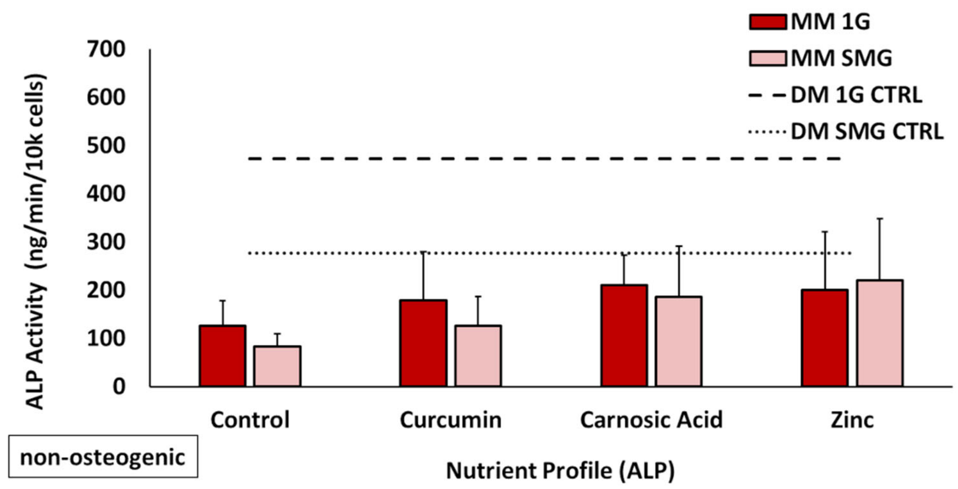

Supplemental Figure S2. Nutraceutical induced osteogenic differentiation in maintenance media (MM). The concentrations of individual nutraceuticals used were based on earlier data (see Supplemental Figure S1): 7.5μM curcumin, 10μM carnosic acid, and 50μM zinc. Lines are imposed are for comparative purposes: osteogenic differentiation media (DM) controls, for 1G and Microgravity. Asterisk (*) $p < 0.05$, (**) $p < 0.01$, (***) $p < 0.001$. The results reflect means ±SD from three independent experiments (n = 3 biological replicates). Statistical analysis was conducted using ANOVA followed by Tukey's post-hoc test.
